# Gamified renal point-of-care ultrasound to promote recruitment and anatomical competence in nephrology

**DOI:** 10.1093/ckj/sfag030

**Published:** 2026-02-17

**Authors:** Philipp Russ, Jonas Einloft, Simon Bedenbender, Martin C Hirsch, Andre Ganser, Ivica Grgic

**Affiliations:** Department of Internal Medicine and Nephrology, Marburg University, University Hospital Giessen and Marburg, Marburg, Germany; Institute for Artificial Intelligence in Medicine, Marburg University, Marburg, Germany; Department of Internal Medicine and Nephrology, Marburg University, University Hospital Giessen and Marburg, Marburg, Germany; Department of Internal Medicine and Nephrology, Marburg University, University Hospital Giessen and Marburg, Marburg, Germany; Institute for Artificial Intelligence in Medicine, Marburg University, Marburg, Germany; Department of Internal Medicine and Nephrology, Marburg University, University Hospital Giessen and Marburg, Marburg, Germany; Department of Internal Medicine and Nephrology, Marburg University, University Hospital Giessen and Marburg, Marburg, Germany; Institute for Artificial Intelligence in Medicine, Marburg University, Marburg, Germany

To the Editor,

Nephrology faces ongoing recruitment challenges, partly driven by ‘nephrophobia’, the perception of the specialty as overly complex and intimidating [1]. Early, clinically contextualized exposure during medical school may help reduce these barriers by fostering engagement and motivation [2]. In preclinical education, ultrasound offers a practical bridge between anatomy and clinical reasoning and can be effectively integrated into renal-focused modules [3]. Consistent with this educational perspective, a 2024 *Clinical Kidney Journal* strategy paper highlighted educational factors across the training continuum as modifiable levers to strengthen interest in the field [4]. In response to calls for expanded point-of-care ultrasound (POCUS) training in nephrology [5], we developed and evaluated an anatomy-integrated, gamified renal POCUS seminar designed to enhance both anatomical competence and early interest in kidney medicine among preclinical students.

In a single-center evaluation (winter term 2025–2026), 101 second-year medical students participated in a 90-minute anatomy session incorporating a dedicated renal POCUS module within the curriculum. The session included a brief introductory lecture covering renal anatomy, acute kidney injury and the clinical utility of POCUS, followed by supervised small-group hands-on scanning using handheld probes. To promote active involvement, we integrated a low-stakes, team-based competition in which groups competed to obtain longitudinal and transverse renal views for each participant. Outcomes were assessed using an anonymized questionnaire comprising 5-point Likert-scale items (1 = strongly disagree to 5 = strongly agree), pre–post self-ratings, and open-ended feedback. Comparisons of pre–post self-ratings were analyzed using paired *t*-tests. Full methodological details are provided in the Supplementary Methods.

Of the 101 participants, 96 (95%) completed the evaluation (item-level *n* varies). Prior exposure to nephrology and ultrasound was minimal (Supplementary Table 2). Overall acceptance was high, with median Likert ratings of 5 across most items; 98% agreed that theory and practice were seamlessly integrated (Supplementary Fig. 2). The team-based, gamified format was perceived as enriching by 89% of students. Participants also reported greater active involvement compared with traditional seminars (76%) and improved recall of practical steps (81%). Competition-related stress remained low (median = 2), supporting the intended low-stakes design of the session (Fig. 1A). Free-text feedback highlighted the perceived value of hands-on practice and interactivity, describing a relaxed, low-pressure atmosphere characterized by positive team dynamics and supportive instructors (Supplementary Fig. 3). Following the module, 98% of participants reported a clearer understanding of clinical indications for renal ultrasound, and 94% indicated improved recognition of relevant abnormalities (Fig. 1B). In line with these findings, self-reported competence increased significantly, with higher ratings for both the ability to visualize the

kidney and knowledge of renal sonoanatomy (both *P* < .001) (Fig. 1C). Beyond perceived skill gains, the module was associated with a favorable attitudinal shift: 85% reported a more positive perception of nephrology, 72% expressed increased interest, and 59% could envision themselves pursuing a nephrology internship (Supplementary Fig. 4). Qualitative responses supported these results, frequently describing nephrology as more accessible and engaging, and noting emerging interest in nephrology-related research (Fig. 1D).

**Figure 1:**
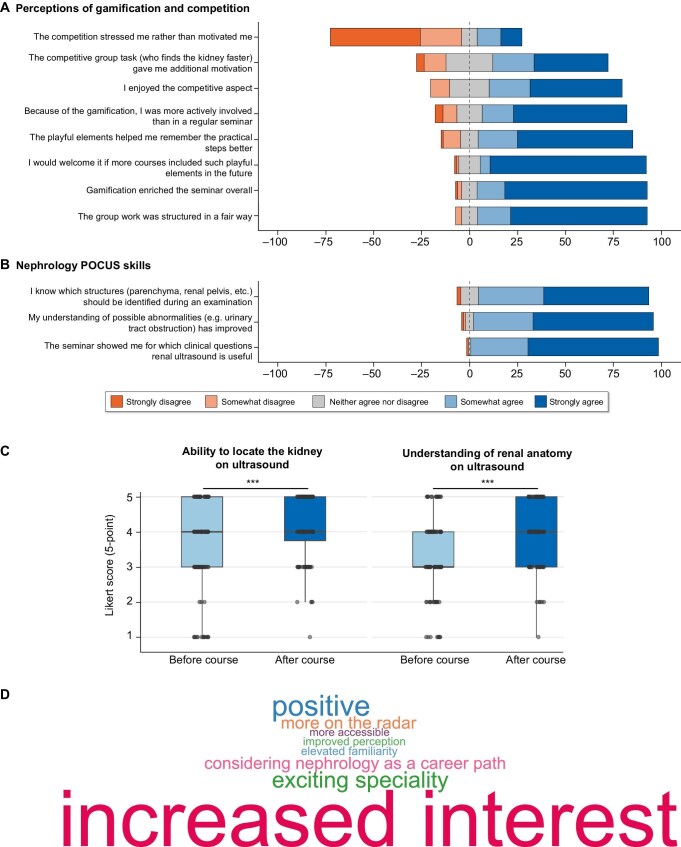
Educational and attitudinal outcomes following a gamified, anatomy-integrated renal POCUS seminar. **(A)** Perceptions of gamification and competition. Diverging stacked bar charts display the distribution of student responses on a 5-point Likert scale (ranging from strongly disagree to strongly agree); values represent percentages (*n* = 98). **(B)** Self-reported nephrology POCUS skills after the seminar, presented as in (A) (*n* = 97). **(C)** Pre–post self-assessments of perceived competence in renal POCUS. Box plots with individual data points show 5-point Likert ratings for the ability to locate and visualize the kidney and for understanding of renal sonoanatomy before and after the seminar (both *P* < .001) (*n* = 97). **(D)** Word cloud of students’ free-text responses describing changes in perceptions of nephrology; word size is proportional to the frequency of mentions (*n* = 35).

Taken together, these findings suggest that an anatomy-integrated, gamified renal POCUS module is feasible within the preclinical curriculum, highly accepted, and associated with short-term gains in perceived competence as well as more favorable attitudes toward nephrology. Limitations include the single-center, uncontrolled design, reliance on self-reported outcomes, potential novelty and ceiling effects and the absence of objective skills assessment or longitudinal follow-up. Accordingly, controlled multicenter and longitudinal studies are needed to assess generalizability, objectively measured competence, and downstream effects on educational and career choices. Nevertheless, early, clinically contextualized renal ultrasound teaching may help reduce perceived barriers (‘nephrophobia’) and strengthen clinically meaningful anatomy learning, while potentially contributing to broader recruitment efforts in nephrology.

## Supplementary Material

sfag030_Supplemental_File
