# Supplementary material for: Gamified renal point-of-care ultrasound to promote recruitment and anatomical competence in nephrology
Source: Clin Kidney J. 2026 Feb 17;19(3):sfag030. doi: 10.1093/ckj/sfag030 (PMC13000338; doi:10.1093/ckj/sfag030)
Supplement: sfag030_Supplemental_File [file sfag030_supplemental_file.docx]

Supplementary Material

**Gamified Renal Point-of-Care Ultrasound to Promote Recruitment and Anatomical Competence in Nephrology**

**Philipp Russ^1,2,*^, Jonas Einloft^1,*^, Simon Bedenbender^1^, Martin Hirsch**^2^**, Andre Ganser^1^, Ivica Grgic^1,2^**^#^

^1^Department of Internal Medicine and Nephrology, Marburg University, University Hospital Giessen and Marburg, Marburg, Germany

^2^Institute for Artificial Intelligence in Medicine, Marburg University, Marburg, Germany

*PR and JE contributed equally to this work

^#^Correspondence:
Ivica Grgic, MD, Department of Internal Medicine and Nephrology,
Marburg University, University Hospital Giessen and Marburg,
Baldingerstrasse 1, 35043 Marburg, Germany.
phone: +49-6421-58-61736
email: grgic@uni-marburg.de
ORCI-ID: [0000-0001-9017-1804](https://orcid.org/0000-0001-9017-1804)

**Table of Contents for the Supplementary Material**

| Supplementary Methods |
| --- |
| Supplementary Figure 1 \| Flowchart of the study design. |
| Supplementary Table 1 \| Design of the questionnaire. |
| Supplementary Table 2 \| Characteristics of the study participants. |
| Supplementary Figure 2 \| General evaluation of the renal POCUS module. |
| Supplementary Figure 3 \| Word cloud of students’ free-text feedback on positive aspects of the seminar design. |
| Supplementary Figure 4 \| Perception of nephrology following the renal POCUS-centered module. |

**Supplementary Methods**

## Participants and Study Design

To evaluate the impact of this innovative nephrology teaching module, we conducted a single-center study involving preclinical, second-year medical students at Marburg University. The study was carried out during the winter term 2025/2026, with the seminar integrated into the anatomy curriculum. Following a 45-minute introductory lecture covering renal anatomy, AKI, and the role and significance of POCUS in nephrology, students were randomly assigned to small teams of eight to nine participants. Each team selected a team name and was provided with one POCUS probe, a display tablet, and supporting materials. An experienced supervisor for available at all times to provide guidance on device handling and to address medical questions.

Within each group, students were tasked with obtaining longitudinal and transverse renal ultrasound views for all team members. Each participant was required both to perform renal ultrasound examinations on another group member and to undergo scanning themselves. For each group, the time from the start of the practical exercise until all students had both performed and received a scan was recorded. The group with the shortest completion time was designated as the fastest thus winning team and received 3D-printed kidney keychains as a symbolic, non-monetary “reward”. A structured debriefing sessions followed, after which participants completed an evaluation questionnaire (Supplementary Figure 1).

## Hardware and Software

Ultrasound examinations were performed using commercially available Butterfly iQ3 handheld probes (Butterfly Network, Burlington, MA, USA), connected via cable to standard tablets (iPad mini-7, Apple, Cupertino, CA, USA) running the Butterfly iQ application (version 2.45.0). All examinations were conducted in B-mode, with ultrasound images displayed in real time on the tablet screens.

## Questionnaire Design

The questionnaire consisted of an initial demographic section with five items assessing age, gender, semester of study, prior POCUS experience, and previous exposure to nephrology. This was followed by separate sections addressing POCUS in general, the gamification component (competitive element), perceived learning gain in nephrology, and perceptions of nephrology as a specialty. Each section included 4–8 items rated on a 5-point Likert scale (1 = strongly disagree, 2 = rather disagree, 3 = neither agree nor disagree, 4 = somewhat agree, 5 = strongly agree). Finally, the questionnaire concluded with four open-ended questions soliciting feedback on the seminar and on innovative teaching concepts. The complete questionnaire is provided in Supplementary Table 1.

## Data Analysis and Graphical Illustration

Survey responses were collected using the LimeSurvey platform (LimeSurvey GmbH, Hamburg, Germany) and exported to Microsoft Excel (version 16, Redmond, WA, USA) for subsequent data handling. Statistical analyses and visualizations were performed using R (version 4.5.0; Vienna, Austria) and RStudio (version 2024.12.1; Boston, MA, USA). Descriptive statistics included means, standard deviations (SD), medians, and interquartile range (IQR) to characterize central tendency and data dispersion. Data are reported as mean ± SD or median with IQR, as appropriate. Given the ordinal nature of Likert-scale responses, non-parametric methods were used. Pre–post comparisons of 5-point Likert-scale items were conducted using the Wilcoxon signed-rank test for paired samples, with effect sizes reported as *r*. The schematic illustration of the study design (Supplementary Figure 1) was created using BioRender (Toronto, Canada).

## Consent to Participate and Ethics Approval

Participants were required to provide informed consent by actively selecting a checkbox before accessing the questionnaire; the survey could not be initiated without consent. All responses were collected and analyzed in anonymized form. Ethical approval for the study was obtained by the Ethics Committee of the Faculty of Medicine, Marburg University (file no. “25-329 ANZ”). All procedures were conducted in accordance with the Declaration of Helsinki and complied with local regulations governing research involving human participants.


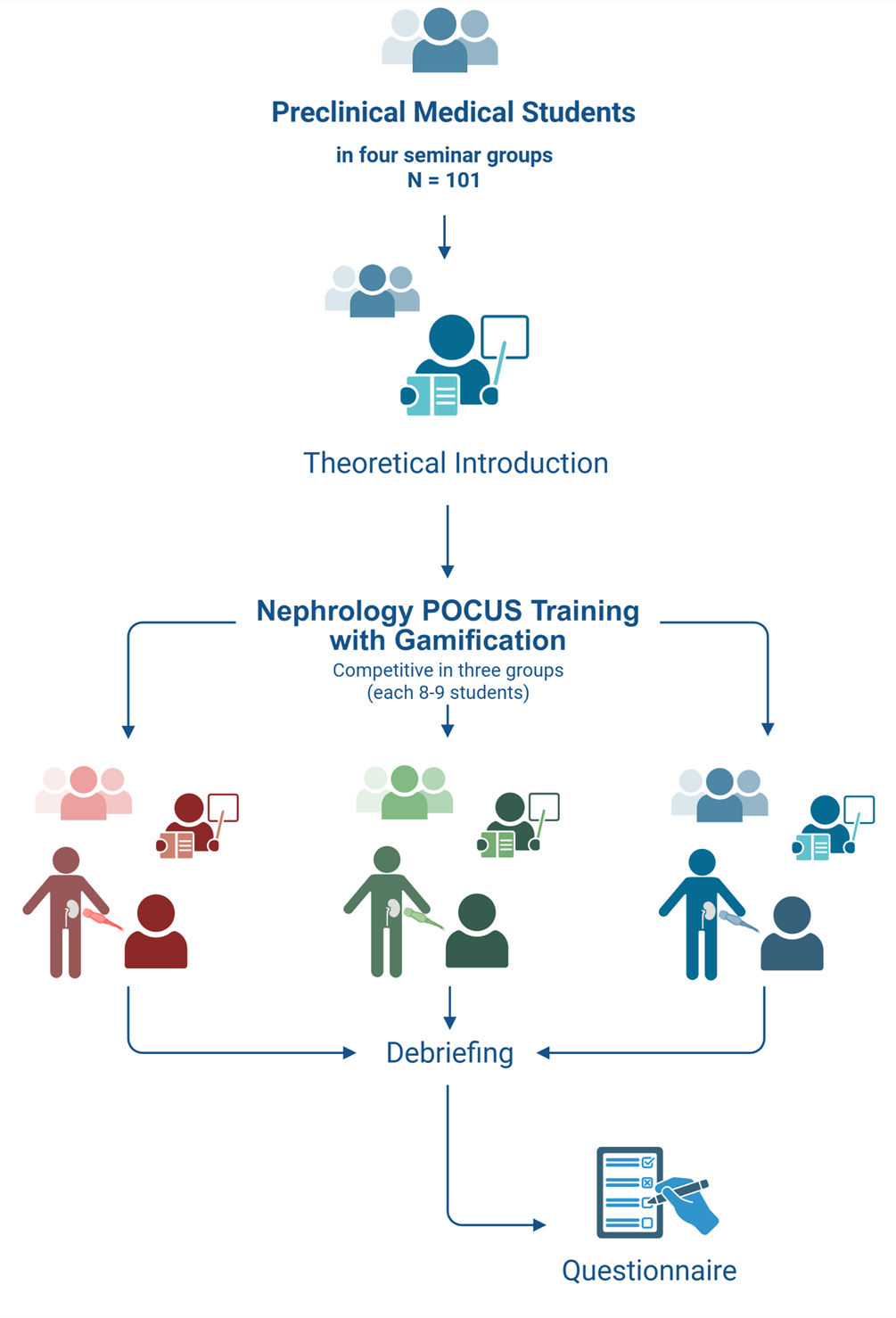


**Supplementary Figure 1 |** Flowchart of the study design. Preclinical medical students (N = 101; four seminar groups of 24-27 participants each) received a theoretical introduction, followed by a hands-on, gamified renal POCUS training session conducted in small competing teams of 8–9 students. The session concluded with a structured debriefing and completion of an evaluation questionnaire.

Supplementary Table 1 | Design of the questionnaire.

| **Question (Number)** | **Question type** | **Question text** |
| --- | --- | --- |
| **Demographics** | Open  Single-choice  Single-choice | Age:  Gender:  Semester: ☐ 3rd semester ☐ 4th semester ☐ other (please specify) |
| **Prior experience** |  |  |
| 1 | Single-choice | Prior POCUS/ultrasound experience before the course:  ☐ none  ☐ one-time demonstration (e.g., during a course/workshop)  ☐ hands-on practice (actively performed scans)  ☐ regularly performed/used (e.g., as a tutor, during a clinical internship) |
| 2 | Single-choice | Previous exposure to nephrology:  ☐ none  ☐ theoretical only (lecture/handouts)  ☐ clinical teaching session / patient case presentation  ☐ personal experience / clinical internship |
| **General Evaluation** |  |  |
| 3 | Likert (5 point) | The practical exercises with the ultrasound device were helpful for my understanding. |
| 4 | Likert (5 point) | The content was tailored to my level of knowledge as a preclinical medical student. |
| 5 | Likert (5 point) | Theory and practice were well integrated. |
| 6 | Likert (5 point) | The handling of the POCUS device was easy for me to follow. |
| 7 | Likert (5 point) | Practical and innovative methods should be used more often in medical education. |
| 8 | Likert (5 point) | The seminar increased my overall motivation for studying medicine. |
| **Perceptions of gamification** |  |  |
| 9 | Likert (5 point) | The competitive group task (who finds the kidney faster) gave me additional motivation. |
| 10 | Likert (5 point) | I enjoyed the competitive aspect. |
| 11 | Likert (5 point) | Because of the gamification, I was more actively involved than in a regular seminar. |
| 12 | Likert (5 point) | The playful elements helped me remember the practical steps better. |
| 13 | Likert (5 point) | The group work was structured in a fair way. |
| 14 | Likert (5 point) | I would welcome it if more courses included such playful elements in the future. |
| 15 | Likert (5 point) | Gamification enriched the seminar overall. |
| 16 | Likert (5 point) | The competition stressed me rather than motivated me. |
| **Competence gain** |  |  |
| 17 | Likert (5 point) | Before the seminar: My understanding of renal anatomy on ultrasound was well developed. |
| 18 | Likert (5 point) | After the seminar: My understanding of renal anatomy on ultrasound was well developed. |
| 19 | Likert (5 point) | Before the seminar: I am generally able to locate the kidney in longitudinal and transverse views on ultrasound. |
| 20 | Likert (5 point) | After the seminar: I am generally able to locate the kidney in longitudinal and transverse views on ultrasound. |
| 21 | Likert (5 point) | The seminar showed me for which clinical questions renal ultrasound is useful. |
| 22 | Likert (5 point) | I know which structures (parenchyma, renal pelvis, etc.) should be identified during an examination. |
| 23 | Likert (5 point) | My understanding of possible abnormalities (e.g., urinary tract obstruction) has improved. |
| **Perception of nephrology** |  |  |
| 24 | Likert (5 point) | I find it exciting to get to know clinical subjects such as nephrology already in the preclinical phase. |
| 25 | Likert (5 point) | The seminar increased my interest in internal medicine, especially nephrology. |
| 26 | Likert (5 point) | I can imagine doing a nephrology internship or elective course. |
| 27 | Likert (5 point) | The combination of POCUS and competition has positively influenced my view of nephrology. |
| **Qualitative Questions** |  |  |
| 28 | Open-ended | What did you particularly like about the seminar (competitive/gamification) design? |
| 29 | Open-ended | What, if anything, did you find bothersome or less appealing about it? |
| 30 | Open-ended | Has your perception of nephrology changed as a result of the seminar? If so, how? |
| 31 | Open-ended | At what point in medical school do you think POCUS should be introduced, and why? |

Supplementary Table 2 | Characteristics of the study participants. Data are presented as counts and percentages; age is reported as mean (SD) and distribution.

| ***Self-reported gender – No.*** | | |
| --- | --- | --- |
| Female | 63 | 62.4% |
| Male | 37 | 36.6% |
| Not specified | 1 | 1% |
| ***Age – years*** | | |
| Mean (standard deviation) | 21.8 (± 2.5) | - |
| Min < Median < Max | 19 < 21 < 35 | - |
| ***Prior POCUS/ultrasound experience before the seminar*** | | |
| none | 58 | 57.4% |
| one-time demonstration (e.g., during a course) | 34 | 33.7% |
| actively practiced (hands-on) | 8 | 7.9% |
| regularly applied (e.g., as a tutor, during a clinical elective) | 1 | 1% |
| ***Previous exposure to nephrology*** | | |
| none | 25 | 24.8% |
| theoretical only (lecture/handout) | 56 | 55.4% |
| clinical session / patient case presentation | 2 | 2% |
| personal experience / clinical internship | 18 | 17.8% |


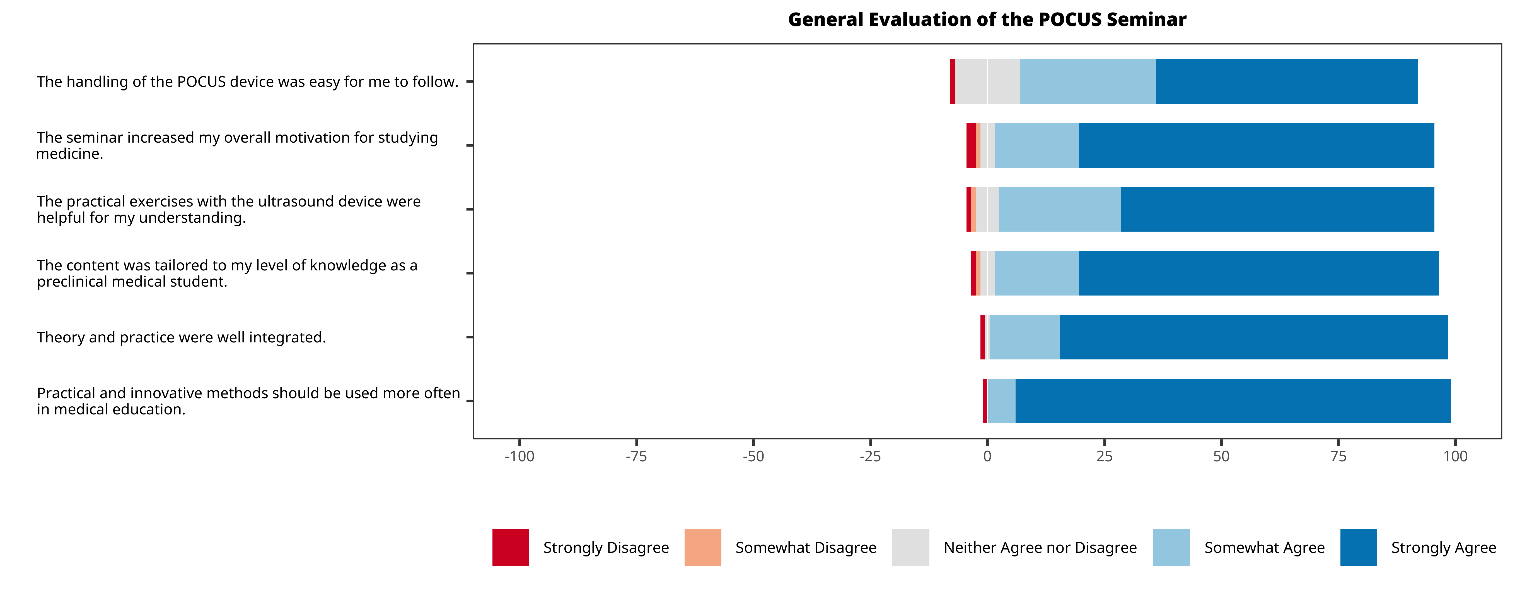


**Supplementary Figure 2.** General evaluation of the renal POCUS module. Diverging stacked bar charts show the distribution of student responses on a 5-point Likert scale (strongly disagree to strongly agree); values represent percentages of respondents (n = 100).

**
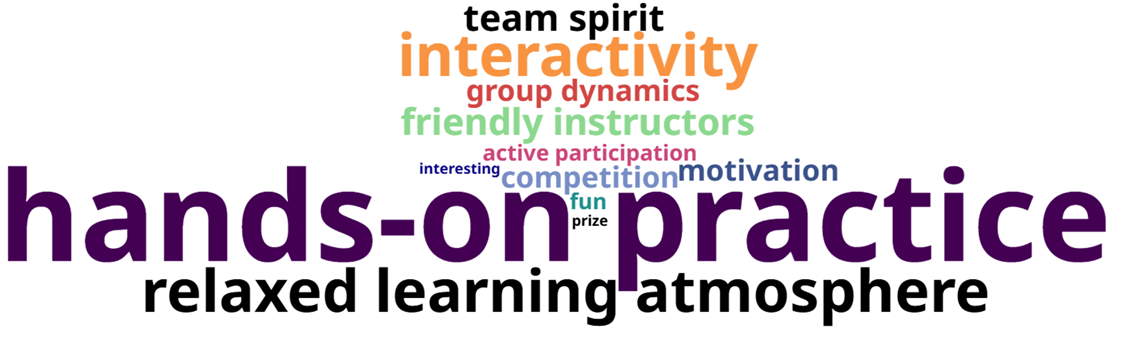
**

**Supplementary Figure 3.** Word cloud of students’ free-text feedback on positive aspects of the seminar design. Word size is proportional to the frequency of mentions (n = 50).


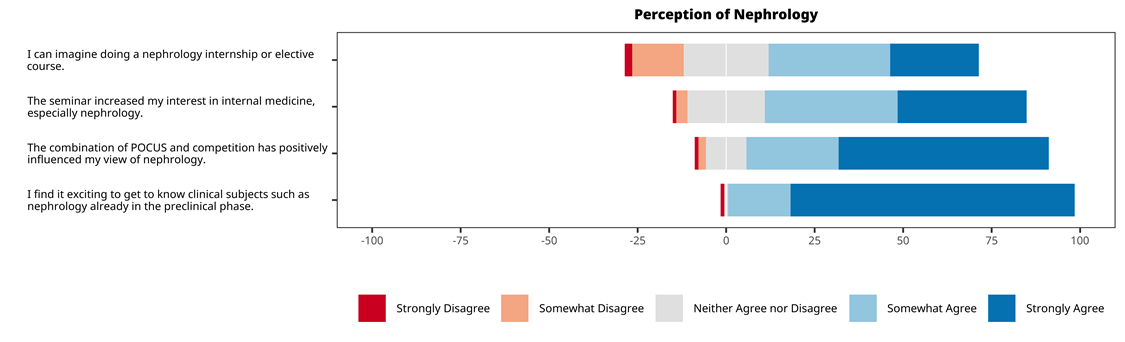
**Supplementary Figure 4.** Perception of nephrology following the renal POCUS-centered module. Diverging stacked bar charts display the distribution of student responses on a 5-point Likert scale (from strongly disagree to strongly agree); values represent percentages of respondents (n = 96).
